# P53 in Penile Squamous Cell Carcinoma: A Pattern-Based Immunohistochemical Framework with Molecular Correlation

**DOI:** 10.3390/cancers15102719

**Published:** 2023-05-11

**Authors:** Isabel Trias, Adela Saco, Lorena Marimon, Ricardo López del Campo, Carolina Manzotti, Oriol Ordi, Marta del Pino, Francisco M. Pérez, Naiara Vega, Silvia Alós, Antonio Martínez, Leonardo Rodriguez-Carunchio, Oscar Reig, Pedro Jares, Cristina Teixido, Tarek Ajami, Juan Manuel Corral-Molina, Ferran Algaba, María J. Ribal, Inmaculada Ribera-Cortada, Natalia Rakislova

**Affiliations:** 1 Department of Pathology, Hospital Clínic of Barcelona, University of Barcelona, 08036 Barcelona, Spain; itrias@clinic.cat (I.T.); masaco@clinic.cat (A.S.);; 2 Barcelona Institute of Global Health (ISGlobal), University of Barcelona, 08036 Barcelona, Spain; 3 Department of Obstetrics and Gynecology, Hospital Clínic of Barcelona, Universityof Barcelona, 08036 Barcelona, Spain; 4 Faculty of Medicine, University of Vic—Central University of Catalonia (UVic-UCC), 08500 Vic, Spain; 5 Translational Genomic and Targeted Therapeutics in Solid Tumors, Institut d’Investigacions Biomèdiques August Pi i Sunyer (IDIBAPS), 08036 Barcelona, Spain; 6 Department of Medical Oncology, Hospital Clínic of Barcelona, 08036 Barcelona, Spain; 7 Uro-Oncology Unit, Hospital Clínic of Barcelona, University of Barcelona, 08036 Barcelona, Spain; 8 Department of Pathology, Fundació Puigvert, Universitat Autònoma de Barcelona, 08025 Barcelona, Spain

**Keywords:** p53 immunohistochemistry, *TP53* mutations, penile squamous cell carcinoma, surrogate marker, pattern-based framework

## Abstract

**Simple Summary:**

Penile squamous cell carcinomas harbouring mutations of *TP53* have an increased risk of lymph node metastases and an impaired prognosis, but the mutational analysis of the *TP53* gene is not available in many pathology laboratories. Although p53 immunohistochemistry (IHC) has been proposed as an alternative to the molecular analysis, the current method of evaluation of p53 IHC has many inaccuracies. The aim of our study was to determine, in a series of 40 penile tumours, if a recently described pattern-based framework of p53 IHC evaluation correlates better than the classical method with the *TP53* mutational status. Our results show that the new method has a very good correlation with *TP53* mutations (95% sensitivity; 92% specificity), higher than that of the classical method, and can be considered as a reliable surrogate of the *TP53* mutational status. This new framework can help clinicians to better define risk groups and refine treatment strategies.

**Abstract:**

p53 immunohistochemistry (IHC) has been proposed as a surrogate for *TP53* mutations in penile squamous cell carcinomas (PSCC). We aimed to evaluate the performance of a pattern-based evaluation of p53 IHC in PSCC. Human papilloma virus (HPV) DNA testing, p16 and p53 IHC, and whole exome sequencing were performed in a series of 40 PSCC. p53 IHC was evaluated following a pattern-based framework and conventional p53 IHC evaluation. Out of 40 PSCC, 12 (30.0%) were HPV-associated, and 28 (70.0%) were HPV-independent. The agreement between the p53 IHC pattern-based evaluation and *TP53* mutational status was almost perfect (k = 0.85). The sensitivity and accuracy of the pattern-based framework for identifying *TP53* mutations were 95.5% and 92.5%, respectively, which were higher than the values of conventional p53 IHC interpretation (54.5% and 70.0%, respectively), whereas the specificity was the same (88.9%). In conclusions, the pattern-based framework improves the accuracy of detecting *TP53* mutations in PSCC compared to the classical p53 IHC evaluation.

## 1. Introduction

Penile squamous cell carcinoma (PSCC) is an unusual neoplasm, with incidence rates that range from 0.5 to 1.6 per 100,000 inhabitants in different European regions [1]. Several risk factors have been identified as possibly implicated in the development of PSCC, including local chronic inflammatory conditions and sexual behaviour, especially exposure to human papillomavirus (HPV) [2].

Two distinct pathways seem to be involved in the carcinogenesis of PSCC: one driven by HPV (HPV-associated) and another independent of HPV infection (HPV-independent) [1]. In keeping with this etiological categorization, the current version of the World Health Organization (WHO) classification of urological tumours [2] divides PSCC according to the presence or absence of HPV. As a consequence, the use of immunohistochemical (IHC) staining for p16, a surrogate marker of the presence of HPV, has become a recommended biomarker to accurately classify these tumours [3]. In Western Europe, a marked predominance of HPV-independent and a low frequency of HPV-associated tumours (70% vs. 30%) has been reported in most studies [4].

The molecular mechanisms involved in HPV-associated PSCC are characterized by genomic instability secondary to the overexpression of the oncoproteins E6 and E7, which lead to uncontrolled activation of the cell cycle [5,6]. In contrast, the pathogenesis of HPV-independent PSCC is less well understood [1]. *TP53* mutations [7,8,9,10] have frequently been reported in this HPV-independent subset of PSCC. Moreover, several studies [11,12] have suggested that *TP53* mutations might be associated with a high frequency of nodal metastases, a factor known to be strongly correlated with impaired prognosis [13,14]. These studies have suggested that *TP53* mutational status could add relevant prognostic information in PSCC [8,11]. However, molecular analysis of the *TP53* gene is technically challenging and not available in many pathology laboratories. Thus, the evaluation of p53 IHC has been proposed as a more feasible alternative [15]. Although a few studies have shown a correlation between p53 IHC overexpression and impaired prognosis in PSCC [16,17], unfortunately, there is a lack of standardization in the evaluation of p53 IHC. Indeed, in most published studies, the assessment of p53 IHC has been based on the percentage of positive nuclei at the basal and parabasal layers [15], and p53 is considered abnormal when diffuse overexpression is identified. However, the cut-off levels have not been clearly established, and thus, the actual meaning of these percentages is not known. Not surprisingly, the studies analysing the correlation between p53 IHC expression and *TP53* mutational status in PSCC have shown discrepant results [18].

Interestingly, the histological features of PSCC as well as the two pathogenic pathways are very similar to the pathology and etiopathogenesis of vulvar carcinomas [19]. Recently, a well-defined, pattern-based framework of p53 IHC evaluation showing a close correlation with *TP53* mutational status has been described in vulvar tumours [20,21]. This pattern-based framework includes four main abnormal p53 IHC patterns that strongly correlate with *TP53* mutations and two normal patterns that reflect a wild-type protein [20,21]. Moreover, based on a combination of HPV status and p53 IHC, three prognostic subtypes of tumours have recently been identified in vulvar squamous cell carcinomas [22].

Due to the marked similarities between PSCC and vulvar carcinoma [23], we hypothesized that this pattern-based framework of p53 IHC interpretation [21] could also be applied to PSCC and have similar implications to those defined in the vulva. Thus, we aimed to explore the correlation between p53 IHC evaluated as described in vulva [21] and the *TP53* mutational status in a series of PSCC from a single institution in Spain, comparing its performance with the conventional interpretation of p53 IHC that generally includes only diffuse overexpression.

## 2. Material and Methods

### 2.1. Case Selection

All PSCCs diagnosed and surgically treated at the Hospital Clinic de Barcelona from 2000 to 2021 were retrieved, and all the available material was reviewed. The initial inclusion criteria for this study were: (1) the presence of invasive PSCC, (2) sufficient material available for HPV testing and IHC evaluation, and (3) available tissue for whole-exome sequencing of the invasive carcinoma. The study was approved by the Ethics Committee of the Hospital Clinic de Barcelona (ref HCB/2020/1207).

A total of 51 PSCC complied with the initial inclusion criteria.

### 2.2. Histological Revision

All of the haematoxylin and eosin sections of the 51 tumours were carefully reviewed. The histological revision aimed to confirm the presence of invasive carcinoma, which was further classified according to the 2022 WHO classification of tumours of the urinary system and male genital organs [2].

In the histological review, a block of formalin-fixed, paraffin-embedded tissue including both the PSCC, and the adjacent skin was selected for HPV testing and IHC staining. In this evaluation, two blocks were also selected for whole-exome sequencing, one representative of the invasive tumour, containing at least 50% of tumour cells (tumour purity estimated by morphology), and another of normal skin or a reactive lymph node, which was selected as control tissue.

### 2.3. HPV Testing, p16 IHC, and Criteria for Classifying a Tumour as HPV-Associated or HPV-Independent

DNA extraction was performed on 10-µm whole tissue sections using a commercial kit (QIAamp DNA FFPE Tissue Kit; Qiagen, Hilden, Germany) as previously described [24]. HPV DNA genotyping was performed using the short polymerase chain reaction (PCR) fragment (SPF10) amplified through the INNO-LiPA HPV Genotyping Extra II Amplification (Fujirebio, Gent, Belgium) [25].

All IHC analyses were performed with the Roche platform. p16 IHC was conducted with the CINtec Histology Kit (clone E6H4). Only the “block” staining pattern with diffuse and intense positivity (nuclear and cytoplasmic) in a group of contiguous cells located in basal and parabasal layers, except in the areas of keratosis and parakeratosis, were considered positive [2].

To categorise a tumour as HPV-associated or -independent, both p16 IHC staining and HPV testing were considered. Any tumour showing a positive p16 IHC and/or HPV DNA testing result was considered as HPV-associated. Tumours negative for both techniques were considered as HPV-independent [2].

### 2.4. p53 IHC Staining and Evaluation

**Figure 1:**
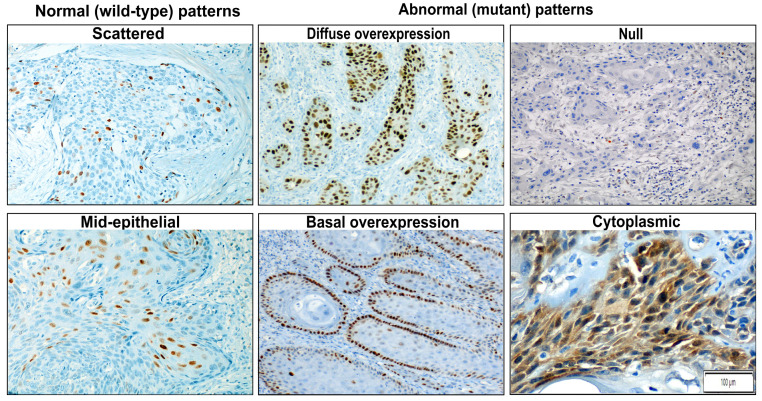
Examples of the six evaluated patterns of p53 immunohistochemical expression in penile squamous cell carcinoma: two normal (wild-type) patterns include scattered and mid-epithelial pattern. Four abnormal (mutant) patterns comprise basal (continuous, strong staining of the nuclei in the basal layer) and diffuse overexpression (continuous and strong nuclear basal staining with suprabasal extension) patterns, null pattern (complete absence of staining in the tumour with positivity in the background inflammatory and stromal cells), and cytoplasmic pattern (with or without nuclear staining).

p53 IHC was performed with the anti-p53 (DO-7) monoclonal antibody (Roche, Vienna, Austria), a widely used antibody that detects both wild-type and mutant p53 [21,22]. Staining in the invasive tumour was evaluated according to the p53 pattern-based interpretation framework recently described for vulvar tumours [21,26]. This framework consists of six patterns grouped into two major categories: normal and abnormal. The “normal” category, which is suggestive of wild-type protein, includes two patterns: (1) occasional positive nuclei in the basal and/or parabasal layer (scattered pattern), and (2) moderate to strong nuclear p53 IHC staining in the parabasal layers with an absence of expression in the basal cells (mid-epithelial pattern). The “abnormal” category, suggestive of mutant protein, includes four p53 IHC staining patterns: (1) continuous, strong nuclear staining of the basal layer (basal overexpression pattern), (2) continuous and strong nuclear basal staining with suprabasal extension of the positive cells (diffuse overexpression pattern), (3) cytoplasmic staining with or without nuclear positivity (cytoplasmic pattern), and (4) complete absence of staining in the tumour, with evidence of intrinsic positive control in the adjacent skin, stromal, or inflammatory cells (null pattern). Figure 1 shows a representative example of the six staining patterns of PSCC.

p53 IHC slides were independently evaluated by three pathologists (I.T., C.M. and N.R.) blind to the molecular results. All pathologists were asked to assign the p53 IHC status (normal vs. abnormal) and pattern for each PSCC. Discordant cases were reviewed between the three evaluators in a meeting where consensus was reached.

In addition to this six-pattern framework, we evaluated the performance of the conventional interpretation of p53 IHC (only diffuse overexpression considered as abnormal) conducted blindly to the molecular results by a fourth pathologist (A.S.) not involved in the evaluations described above.

### 2.5. Whole Exome Sequencing and Bioinformatic Analysis

DNA was isolated as described above from invading tumour and matched normal tissue (skin or lymph node). For the DNA isolation, between 20 and 200 ng of gDNA were sheared using a Covaris™ LE220-Plus (MA, USA) and underwent quality control on an Agilent 2100 Bioanalyzer (CA, USA). The adaptor-modified end library was amplified by 10, 15 or 18 cycles of pre-capture PCR with the 2x KAPA HiFi HotStart ReadyMix PCR Kit (Roche). Pools of eight indexed libraries of a combined mass of 1.5 microgram were set up for hybridization (55 °C; 16 h). After washes, the pooled libraries were PCR-amplified. The libraries were sequenced on NovaSeq 6000 (Illumina, San Diego, CA, USA) in paired-end mode with a read length of 2 × 151 bp.

Reads were mapped to the human genome (hs37d5) using the Burrows-Wheeler Alignment and processed using Picard tools version 1.110. The Genome Analysis Tool Kit [27] was used for local indel realignment and base recalibration. Somatic variant calling was performed with GATK v4.1.9.0 Mutect2 and Strelka2 v2.8.3, and annotation with SnpEff v.4.3.e and SnpSift. Copy number variants were predicted with Control-FREEC [28] and annotated with SnpEff.

Cases with tumour and/or non-tumour samples with low coverage depth (<20×) were excluded from the analysis.

### 2.6. Attribution of TP53 Mutational Status

*TP53* was considered mutated if a somatic mutation/s and/or loss in copy number were identified. Only the variants with an allele frequency >4% [29], predicted by both the Mutect2 and Strelka2 databases, and that had passed the quality filters of each program were considered as driver mutations. Tumours with no identified *TP53* somatic mutations or variants with allele frequency <4%, and tumours showing only gains in the *TP53* gene, in the absence of somatic mutations, were classified as *TP53* wild-type [30]. The pathogenicity level (clinical significance) for each identified *TP53* somatic variant was retrieved from the National Centre of Biotechnology Information ClinVar database [31].

### 2.7. Statistical Analysis

StataC/v15.0.591 (StataCorp, College Station, TX, USA) was used for all the data analyses. The clinical and histopathological data were compared using Chi-square tests (categorical data) and analysis of variance (numerical data). The diagnostic test performance of p53 IHC evaluation against *TP53* mutational status (gold standard), as well as inter-observer agreement, was calculated using the Fleiss’ kappa test. The strength of agreement of kappa values was evaluated following the Landis-defined categories: 0, none beyond chance; 0–0.20, slight; 0.21–0.40, fair; 0.41–0.60, moderate; 0.61–0.80, substantial; and 0.81–1.00, almost perfect [32]. The sensitivity, specificity, positive and negative predictive values, and accuracy of p53 IHC evaluation, with 95% confidence intervals (95%CI), were also calculated. A two-sided *p*-value < 0.05 was considered statistically significant.

## 3. Results

### 3.1. Cases Included in the Study and Association with HPV

Eleven tumours were excluded from the study due to insufficient coverage depth (<20×) of the tumour or the matched control tissue (8 and 3 samples, respectively). Forty tumours fulfilled the inclusion criteria and were included in the study. Twelve out of the 40 PSCC (30.0%) were classified as HPV-associated PSCC, with seven cases showing p16 overexpression and HPV-DNA and five cases being only positive for p16 IHC with a negative HPV testing result. In all seven HPV-DNA positive cases, HPV16 was the only type identified. Twenty-eight out of 40 tumours (70.0%) were negative for p16 and HPV testing and were classified as HPV-independent.

### 3.2. Characteristics of the Overall Series

Table 1 shows the main clinic-pathological characteristics of the PSCC patients, categorised into the two major groups, HPV-associated and HPV-independent, as well as their stage at diagnosis. Patients with HPV-associated PSCC were slightly younger (mean 65.2 years; range 45–94) than the HPV-independent patients (mean 69.1 years; range 40–86) (*p* = 0.23).

### 3.3. TP53 Mutations

The mean percentage of tumoral cells in the samples (tumour purity) was 58% (range 0.50–0.89). The average tumour sequencing depth was 81×, ranging from 23× to 314×. *TP53* mutations (somatic and/or copy number alterations) were identified in 22 PSCC (52.5%), whereas 18 tumours (47.5%) were *TP53* wild-type. Nineteen PSCC harboured 21 somatic variants, with two of them additionally showing *TP53* copy number alterations. Three tumours showed only *TP53* copy number loss. The mean allele frequency of the 21 identified somatic *TP53* variants was 0.21 (range 0.04–0.79). *TP53* missense variants were the most prevalent (14/21; 66.6%), followed by nonsense (4/21; 19.0%), splice-site (2/40; 5.0%) and frameshift (1/4; 2.5%).

### 3.4. Agreement between p53 IHC and TP53 Mutational Status and Interobserver Agreement

**Figure 2:**
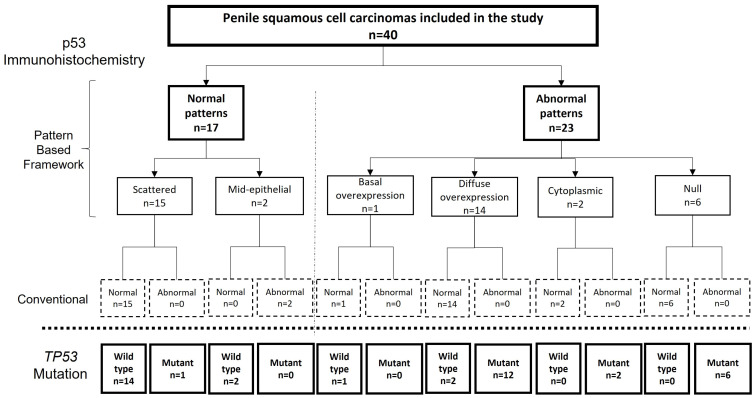
Flow chart showing the results of the pattern-based framework evaluation of p53 immunohistochemistry (IHC), their correlation with the classic IHC evaluation of p53 and with the results of the genetic analysis.

The agreement between the p53 IHC status and *TP53* mutational status was substantial for two observers, and moderate for the third observer (k = 0.64, 0.75 and 0.50, respectively; *p* < 0.0001 for each). After the consensus meeting, in which the twelve discordant cases were discussed, the agreement between the final p53 IHC pattern-based evaluation and the *TP53* mutational status increased to almost perfect (k = 0.85; 95% CI = 0.68–1). In this consensus evaluation of p53 IHC, 17 tumours (42.5%) were classified as normal. Fifteen of them showed scattered and two mid-epithelial staining. Twenty-three tumours were classified as showing an abnormal p53 IHC pattern. Of these, 14 (60.8%) showed diffuse overexpression, six (26.0%) null pattern, two (8.6%) cytoplasmic staining, and one (4.3%) basal overexpression. All but one tumour assigned as p53 IHC normal were *TP53* wild-type (16/17; agreement: 94.1%), and all but two tumours evaluated as p53 IHC abnormal in the consensus were *TP53*-mutant (21/23; agreement: 91.3%). The agreement of the p53 IHC pattern-based evaluation (normal vs. abnormal) between three observers was moderate (k = 0.59). A flowchart showing the results of the pattern-based framework evaluation of p53 IHC, their correlation with the classic IHC evaluation of p53, and with the results of the genomic analysis of *TP53* are shown in Figure 2. The differences between the pattern-based and the conventional p53 IHC evaluation were related to a misclassification as abnormal expression of the mid-epithelial pattern and a misclassification as normal expression of the cases showing cytoplasmic, null, or basal overexpression.

The sensitivity, specificity, and accuracy of each ratter and of the consensus evaluation, as well as the figures for the evaluation using conventional criteria are shown in Table 2. The final p53 IHC pattern-based evaluation sensitivity, specificity and accuracy were 95.5% (95%CI 77.2–99.9%), 88.9% (95%CI 65.3–98.6%) and 92.5% (95%CI 79.6–98.4%), respectively.

A detailed description of the p53 IHC patterns and *TP53* mutational status of the 40 PSCC and their relationship with HPV status is shown in Table 3.

Frequencies and numbers of *TP53* variants in other type of cancers (found to be discordant with pattern-based p53 IHC evaluation for at least one of the three observers) are shown in the Supplementary Table S1.

### 3.5. p53 IHC Patterns and TP53 Status in HPV-Associated PSCC

Ten out of 12 (83.3%) HPV-associated tumours showed normal p53 IHC pattern. All of them were *TP53* wild-type. Eight tumours showed scattered p53 staining, and two showed a mid-epithelial pattern. In seven tumours, the p53 IHC normal pattern was unanimously assigned by the three observers, whereas in three cases, at least one of the observers diagnosed a p53 IHC abnormal pattern.

**Figure 3:**
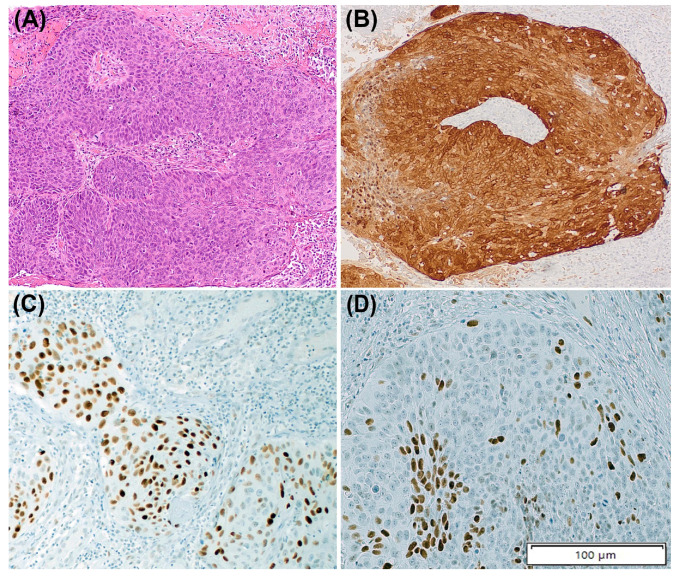
The case of Human Papillomavirus (HPV)-associated penile squamous cell carcinoma (PSCC) with basaloid features (**A**). Immunohistochemistry for p16 is positive (**B**) and p53 staining shows an abnormal pattern of diffuse overexpression (**C**). However, in some areas, the tumour shows scattered p53 staining (**D**). A pathogenic *TP53* nonsense mutation (c.637C>T [R213*]) and a loss in *TP53* copy number have been identified in this tumour.

An abnormal p53 IHC pattern was identified in 2/12 (16.7%) HPV-associated tumours, the two of them showing diffuse p53 overexpression pattern, which was independently assigned by each of the three pathologists. In the sequencing analysis, one tumour showed a pathogenic *TP53* c.637C>T nonsense mutation (p.Arg213Ter) with a variant allele frequency of 0.05 accompanied by a loss in the *TP53* copy number. Figure 3 shows the histological and IHC features of this tumour.

**Figure 4:**
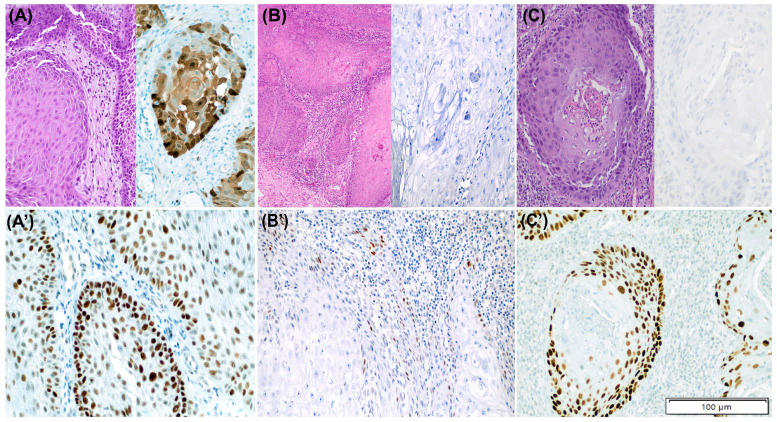
Three discordant cases between p53 immunohistochemistry (IHC) and *TP53* mutational status in penile squamous cell carcinomas (PSCC). (**A**–**C**) show H/E staining together with p16 IHC staining and (**A’**–**C’**) outline p53 IHC staining for each case. (**A**,**A’**): HPV-associated PSCC with p53 abnormal pattern (diffuse overexpression) and no evidence of *TP53* alterations. (**B**,**B’**): HPV-independent tumour with normal (scattered) p53 IHC pattern and presence of likely benign missense *TP53* mutation (c.251C>T [A84V]). (**C**,**C’**): HPV-independent PSCC with p53 abnormal pattern (diffuse overexpression) and no evidence of *TP53* alterations.

Contrarily, in the second case, the exome sequencing did not reveal any *TP53* alteration. Figure 4A,A’ illustrates the latter discordant case. None of the HPV-associated PSCC tumours showed cytoplasmic, null, or basal overexpression pattern.

### 3.6. p53 IHC Patterns and TP53 Status in HPV-Independent PSCC

Seven out of the 28 (25.0%) HPV-independent tumours showed normal p53 IHC; all of them displayed scattered pattern. Six showed *TP53* wild-type status in the sequencing analysis and one harboured a likely benign *TP53* c.251C>T missense mutation (p. Ala84Val), with a variant allele frequency of 0.04. Figure 4B,B’ illustrates the latter case. In four cases, the normal p53 IHC status was assigned by the three observers (full concordance), whereas in three cases, including the *TP53*-mutated case, one of the observers suggested an abnormal p53 pattern. No tumours with mid-epithelial pattern were identified in the HPV-independent PSCC group.

An abnormal p53 IHC pattern was identified in 21/28 (75.0%) HPV-independent tumours. Twelve of them (57.1%) showed a diffuse overexpression pattern, six (28.6%) a null pattern, two tumours (9.5%) showed cytoplasmic and one (4.8%) basal staining. Twenty out of the 21 (95.2%) PSCC with abnormal p53 IHC were *TP53*-mutant. Of them, 17 tumours (85.0%) showed at least one somatic *TP53* alteration and three (15.0%) only *TP53* copy number loss. In 15/21 tumours (71.4%), all three observers assigned p53 abnormal IHC (including a *TP53* wild-type tumour), whereas in six cases, an abnormal p53 IHC was diagnosed by at least one observer. Figure 4C,C’ shows the PSCC with abnormal p53 IHC and absence of *TP53* mutations.

Of the 17 cases with *TP53* somatic mutations and abnormal p53 IHC, 11 (64.7%) harboured at least one pathogenic or likely pathogenic variant, five (29.4%) showed only a variant of uncertain significance and in one (5.9%) case the mutational variant was not found in ClinVar database (frameshift c.273dupT codifying p.Glu131fs protein). Eight out of 10 (80.0%) PSCC with diffuse overexpression pattern were enriched in pathogenic/likely pathogenic variants of *TP53*, followed by those with null pattern (2/4; 50.0%), cytoplasmic pattern (1/2; 50.0%), while the only tumour with basal overexpression harboured the variant of uncertain significance.

Among the three tumours with only *TP53* copy number loss, two showed p53 IHC null pattern and one diffuse overexpression p53 IHC pattern. Finally, the tumour with abnormal p53 IHC and *TP53* wild-type status showed diffuse overexpression (with full concordance between 3 observers).

## 4. Discussion

In this study, we evaluated the correlation between p53 IHC expression and *TP53* mutations in PSCC, using for the first time the pattern-based p53 IHC evaluation framework recently described in vulvar tumours. In keeping with the data reported in the vulva [21,26], this pattern-based p53 IHC evaluation framework reliably predicted the *TP53* mutational status of the PSCC (95.7% sensitivity, 88.9% specificity, 92.5% accuracy).

Several studies have shown that *TP53* mutational status is clinically relevant in patients with PSCC because mutations are associated with an increased risk of lymph node metastases and impaired prognosis [8,10,11,12,13]. However, *TP53* sequencing is technically challenging to implement in the routine. Consequently, several investigators have proposed using p53 IHC staining as a surrogate of *TP53* [14], with conflicting results in the correlation between both techniques [8,18]. This poor correlation can be attributed to several factors. Firstly, previous studies have considered diffuse p53 IHC overexpression as the only abnormal pattern suggestive of *TP53* mutation, which has shown a limited sensitivity in most series [8]. Secondly, there is a lack of standardisation in the evaluation of p53 staining: although p53 IHC assessment is usually based on the percentage of positive nuclei at the basal and parabasal layers [14], the threshold of positivity suggesting mutation has not been clearly defined. While some studies consider as abnormal p53 staining a positivity in at least 20% of the nuclei [33], other investigators have used a combination of intensity and extent of the positivity [34]. Remarkably, the sensitivity and accuracy of the pattern-based framework of p53 IHC expression to detect *TP53* mutation (95.5% and 92.5%, respectively) were much higher than the classical criteria considering only diffuse positive staining as abnormal (9) (54.5% and 70.0%, respectively). However, the specificity did not vary at all between the two methods of evaluation (88.9%).

The correlation between normal p53 IHC using the pattern-based evaluation framework and wild-type *TP53* status was excellent in our study (16/17; 94.1%). Interestingly, the only tumour with normal p53 IHC staining but *TP53*-mutated status harboured a *TP53* mutation classified as likely benign, probably not involved in the pathogenesis of this neoplasm. Thus, if only the pathogenic variants were used to define *TP53*-mutated status, this case would have been reclassified as p53 IHC-*TP53* concordant, which would increase the correlation between normal p53 IHC and *TP53* wild-type status to 100%. The two p53 IHC patterns described as normal in vulvar carcinomas, scattered and mid-epithelial pattern [21], were identified in our series. The mid-epithelial pattern, previously described in HPV-associated vulvar cancers [21,26,35,36], and identified in two HPV-associated PSCC, is of particular interest. This type of staining probably reflects senescence of high-risk HPV-infected neoplastic cells and represents a potential diagnostic pitfall with p53 overexpression [36], if the pathologist only notices the strong staining in the centre of the tumoral nests.

The excellent correlation between normal p53 IHC and wild-type *TP53* status is in contrast with the data reported by Kashofer et al. [8], who, applying the conventional p53 IHC evaluation criteria, showed high frequency of *TP53*-mutated tumours with normal p53 staining. It should be noted that some of the p53 patterns considered as abnormal in the pattern-based p53 IHC evaluation framework, especially the cytoplasmic and the null patterns, are considered as normal in the conventional evaluation.

We also observed an excellent correlation between abnormal p53 IHC using the pattern-based evaluation framework and *TP53*-mutant status (21/23; 91.3%). However, if only pathogenic or likely pathogenic variants were considered to define *TP53* mutant status, the correlation would drop to 65.2% (15/23). The most frequent abnormal p53 IHC pattern was diffuse overexpression (61%), the only pattern previously considered as abnormal in previous studies on PSCC [8]. It is also the most frequent pattern in vulvar tumours [20,21,26], stomach [37], and ovary [38], usually associated with a missense *TP53* mutation. In addition to this common pattern, two additional abnormal patterns of p53 IHC expression (null and cytoplasmic) were identified in as many as one-third of HPV-independent tumours. These two patterns have been identified in HPV-independent vulvar tumours [21,39] but, to our knowledge, have not been previously described in PSCC. Finally, basal overexpression pattern was the most uncommon pattern in our series, observed in only one tumour. As reported in the vulva [21] the distinction between wild-type expression and basal overexpression is often challenging. In addition, this case had a *TP53* variant with uncertain pathogenicity and thus might as well be classified as wild-type if only pathogenic variants had been used to define *TP53*-mutated status.

The findings of our study are consistent with previously reported data [29], showing that *TP53* mutations are much more frequent in HPV-independent than in HPV-associated PSCC [7]. Indeed, 75% of the HPV-independent and 8.3% of the HPV-associated PSCC in our series had *TP53* alterations (*p* < 0.001). These differences were also observed for p53 IHC using the pattern-based framework: abnormal patterns were identified in 75% of the HPV-independent and 16.6% of the HPV-associated PSCC (*p* = 0.005).

The case of HPV-associated tumour with diffuse p53 overexpression and a pathogenic nonsense *TP53* variant (c.637C>T codifying for p.Arg213Ter protein) is certainly interesting, as *TP53* mutations are highly uncommon in HPV-related neoplasm [7,29,40]. Remarkably, the same mutational variant of *TP53* was identified in two additional cases in our series, both HPV-independent, one with diffuse overexpression and one with null p53 pattern. The mutation has been reported to cause a truncated or absent *TP53* protein [31], thus correlating with diffuse overexpression and null IHC patterns identified in our study, respectively. The variant was occasionally reported in PSCC [7,41] but was never correlated with p53 IHC staining.

The main strength of our study is that we analysed the *TP53* gene by exome sequencing, targeting both somatic mutations and copy number alterations, which has allowed us to obtain an accurate correlation between the findings of IHC and the molecular analysis. In addition, we have used a well-defined pattern-based framework of p53 IHC evaluation that has shown a good correlation in vulvar squamous cell carcinoma, a neoplasm with similar etiopathogenic background [21]. This framework allowed the identification of several abnormal patterns of expression not previously identified in PSCC and showed better correlation with the *TP53* molecular status than the conventional criteria. The main limitation is the small sample size, particularly of HPV-associated tumours, which precludes obtaining a reliable distribution of p53 IHC patterns. A second limitation is the use of formalin-fixed, paraffin-embedded tissue for sequencing, which might have resulted in under- or over-identification of *TP53* alterations. Finally, the tumour purity of the samples was relatively low (58%), which could have impacted the somatic variant calling conducted in this study. Lastly, although we obtained strong correlation between p53 IHC and mutational status, we identified a proportion of tumours with *TP53* variants of uncertain significance, which introduced a challenge in attribution of *TP53* mutational status.

## 5. Conclusions

In conclusion, our study shows that the pattern-based framework of p53 IHC evaluation accurately predicts *TP53* mutational status in PSCC, improving the performance of previously reported methods of p53 IHC evaluation. This new framework recognises three new patterns (mid-epithelial, null and cytoplasmic) in PSCC that would be misclassified by conventional criteria, while the existence of basal pattern is questionable. Further molecular studies are warranted to validate our findings in larger cohorts of PSCC.

## Figures and Tables

**Table 1:** Clinical and pathological characteristics of the penile squamous cell carcinomas (PSCC) included in the study categorized in the two main pathological types.

	HPV-Associated PSCC (*n* = 12)	HPV-Independent PSCC (*n* = 28)	*p*
**Mean age (years) ± standard deviation**	65.2 ± 14.9	69.1± 13.1	0.23
**Histological subtype**			<0.001
Usual/verrucous	3 (25.0%)	27 (96.4%)	
Basaloid/warty/lymphoepithelioma-like	9 (75.0%)	1 (3.6%)	
**Vascular invasion**	1 (8.3%)	3 (10.7%)	0.8
**Perineural invasion**	1 (8.3%)	2 (7.1%)	0.8
**Lymph node metastases**	3 (25.0%)	8 (28.6%)	0.8
**Stage**			1
Initial (I)	6 (50.0%)	14 (50.0%)	
Advanced (II/III/IV)	6 (50.0%)	14 (50.0%)	
**Treatment**			1
Surgery	10 (83.3%)	21 (75.0%)	
Surgery + radiation and/or chemotherapy	2 (16.6%)	7 (25.0%)	

HPV: human papillomavirus.

**Table 2:** The p53 immunohistochemical (IHC) evaluation in penile squamous cell carcinomas (PSCC) for each of the observers and after consensus meeting.

	p53 Immunohistochemistry Evaluation										
	Pattern-Based *								Conventional **		*TP53* Status
	Observer 1		Observer 2		Observer 3		Consensus		Observer 4		
	Normal	Abnormal	Normal	Abnormal	Normal	Abnormal	Normal	Abnormal	Normal	Abnormal	
*TP53* wild-type	15	3	14	4	13	5	16	2	16	2	18
*TP53* mutant	2	20	6	16	2	20	1	21	10	12	22
Total	17	23	20	20	15	25	17	23	26	14	40
Sensitivity	90.9 (70.8–98.9)		72.7 (49.8–89.3)		90.9 (70.8–98.9)		95.5 (77.2–99.9)		54.5 (32.2–75.6)		
Specificity	83.3 (58.6–96.4)		77.8 (52.4–93.6)		72.2 (46.5–90.3)		88.9 (65.3–98.6)		88.9 (65.3–98.6)		
Accuracy	87.5 (73.2–95.8)		75.0 (58.8–87.3)		82.5 (67.2–92.7)		92.5 (79.6–98.4)		70.0 (53.5–83.4)		

Sensitivity, specificity, and accuracy are shown as percentage and (95% confidence interval); * The pattern-based evaluation considers two normal p53 IHC patterns (scattered and mid-epithelial) and four abnormal patterns (null, cytoplasmic, basal overexpression and diffuse overexpression); ** The conventional evaluation includes only diffuse overexpression as abnormal, and all other types of staining are classified as normal.

**Table 3:** Summary of immunohistochemical (IHC) and molecular features for each of the 40 penile squamous cell carcinomas.

Case	HPV Status	p53 IHC Status	p53 IHC Pattern	*TP53* Status	*TP53* Variant (Protein Change)	Type of *TP53* Mutation	VAF	Clinical Significance of *TP53* Variant	*TP53* Copy Number Status
1	Pos	Normal	Scatt	Wt	-	-	-	-	Normal
2	Pos	Normal	Scatt	Wt	-	-	-	-	Normal
3	Pos	Normal	Scatt	Wt	-	-	-	-	Normal
4	Pos	Normal	Scatt	Wt	-	-	-	-	Normal
5	Pos	Normal	Scatt	Wt	-	-	-	-	Normal
6	Pos	Normal	Scatt	Wt	-	-	-	-	Normal
7	Pos	Normal	Scatt	Wt	-	-	-	-	Gain
8	Pos	Normal	Scatt	Wt	-	-	-	-	Gain
9	Pos	Normal	Mid-ep	Wt	-	-	-	-	Normal
10	Pos	Normal	Mid-ep	Wt	-	-	-	-	Normal
11	Pos	Abnormal	Diff OE	Mut	c.637C>T (R213*)	Nonsense	0.05	Pathogenic	Loss
12	Pos	Abnormal	Diff OE	Wt	-	-	-	-	Normal
13	Neg	Normal	Scatt	Wt	-	-	-	-	Normal
14	Neg	Normal	Scatt	Wt	-	-	-	-	Normal
15	Neg	Normal	Scatt	Wt	-	-	-	-	Normal
16	Neg	Normal	Scatt	Wt	-	-	-	-	Normal
17	Neg	Normal	Scatt	Wt	-	-	-	-	Normal
18	Neg	Normal	Scatt	Mut	c.251C>T (A84V)	Missense	0.04	Likely benign	Normal
19	Neg	Normal	Scatt	Wt	-	-	-	-	Gain
20	Neg	Abnormal	Null	Mut	-	-	-	-	Loss
21	Neg	Abnormal	Null	Mut	-	-	-	-	Loss
22	Neg	Abnormal	Diff OE	Mut	-	-	-	-	Loss
23	Neg	Abnormal	Diff OE	Wt	-	-	-	-	Normal
24	Neg	Abnormal	Basal	Mut	c.530C>T (P177L)	Missense	0.27	Likely pathogenic/uncertain	Normal
25	Neg	Abnormal	Cyt	Mut	c.574G>A (A192T)	Missense	0.04	Uncertain	Normal
26	Neg	Abnormal	Diff OE	Mut	c.1082G>A (G361E)	Missense	0.07	Uncertain	Normal
27	Neg	Abnormal	Diff OE	Mut	c.919+1G>A (-)	Splice-site	0.06	Likely pathogenic	Normal
28	Neg	Abnormal	Diff OE	Mut	c.527G>T (C176F)	Missense	0.12	Pathogenic/likely pathogenic	Normal
29	Neg	Abnormal	Diff OE	Mut	c.843C>G (D281E)	Missense	0.16	Uncertain/likely pathogenic	Normal
30	Neg	Abnormal	Cyt	Mut	c.991C>T (Q331*)	Nonsense	0.11	Pathogenic	Normal
31	Neg	Abnormal	Diff OE	Mut	c.335G>A (S112N)	Missense	0.79	Uncertain	Normal
32	Neg	Abnormal	Diff OE	Mut	c.524G>A (R175H)	Missense	0.12	Likely Pathogenic/uncertain	Gain
33	Neg	Abnormal	Diff OE	Mut	c.799C>T (R267W)	Missense	0.05	Pathogenic/likely pathogenic	Gain
34	Neg	Abnormal	Diff OE	Mut	c.844C>T (R282W)	Missense	0.26	Pathogenic/likely pahogenic	Normal
35	Neg	Abnormal	Null	Mut	c.637C>T (R213*)	Nonsense	0.32	Pathogenic	Normal
36	Neg	Abnormal	Null	Mut	c.273dupT (-)	Frameshift	0.65	Not found	Normal
37	Neg	Abnormal	Null	Mut	c.817C>T (R273C)	Missense	0.05	Pathogenic/likely pathogenic	Normal
38	Neg	Abnormal	Null	Mut	c.461G>A (G154D); c.276+1G>C (-)	Missense; Splice-site	0.05; 0.64	Uncertain; Not found	Normal
39	Neg	Abnormal	Diff OE	Mut	c.517G>A (V173M); c.637C>T (R213*)	Missense; Nonsense	0.21; 0.1	Pathogenic; Pathogenic	Normal
40	Neg	Abnormal	Diff OE	Mut	c.524G>A (R175H)	Missense	0.44	Likely Pathogenic/uncertain	Normal

Cyt: cytoplasmic; Diff OE: diffuse overexpression; Mid-ep: mid-epithelial; Mut: mutant; Neg: negative; Pos: positive; Scatt: scattered; VAF: variant allele frequency; Wt: wild-type; (-) protein unknown.

## Data Availability

All data used in this study are available and can be accessed upon reasonable request.

## Supplementary Materials

The following supporting information can be downloaded at: https://www.mdpi.com/article/10.3390/cancers15102719/s1, Table S1: Frequencies of identified *TP53* variant among other TP53 mutations and number and types of cancers in which the variant was reported for five penile squamous carcinomas (PSCC) in which there was discordance between p53 immunohistochemistry (IHC) evaluation (for at least one of the three observers) and *TP53* mutational status.
