# Supplementary material for: P53 in Penile Squamous Cell Carcinoma: A Pattern-Based Immunohistochemical Framework with Molecular Correlation
Source: Cancers (Basel). 2023 May 11;15(10):2719. doi: 10.3390/cancers15102719 (PMC10216449; doi:10.3390/cancers15102719)
Supplement: Supplementary file 1 [file cancers-15-02719-s001.zip › cancers-2339167-supplementary.pdf]

Supplementary Table S1: Frequencies of identified *TP53* variant among other *TP53* mutations and number and types of cancers in which the variant was reported for 5 penile squamous carcinomas (PSCC) in which there was discordance between p53 immuno-histochemistry (IHC) evaluation (for at least one of the 3 observers) and *TP53* mutational status.

Type of *TP53* variant (phenotype)	Evaluation of p53 IHC				Genomic database	
	Obs 1	Obs 2	Obs 3	Consensus	TCGA-pancancer studies	COSMIC
c.251C>T, missense (A84V)	wt	wt	mut	wt	0 (0%)	3 (N/A) (2 gliomas, 1 adult T cell lymphoma)
c.1082G>A, missense (G361E)	mut	wt	mut	mut	0 (0%)	2 (N/A) (1 cervix squamous carcinoma, 1 skin melanoma)
c.524G>A, missense (R175H)	mut	wt	mut	mut	150/996 (43%) (32 colonic carcinomas, 21 breast carcinomas, 15 ovary serous carcinomas, 12 head and neck squamous carcinomas, among other less frequent types)	1291 (NA) (709 colonic, 182 breast, 150 oesophageal, 128 gastric and 122 central nervous system tumors)
c.799C>T, missense (R267W)	mut	mut	wt	mut	6/996 (0.6%) (3 brain gliomas, 1 renal carcinoma, 1 uterine carcinomas, 1 colonic carcinoma)	43 (N/A) (14 colonic, 14 central nervous system, 7 liver, 4 thyroid and 4 gastric tumors)
c.817C>T, missense (R273C)	wt	wt	mut	mut	125/996 (12.5%) (58 brain gliomas, 10 serous ovarian carcinomas, 6 head and neck carcinomas, among other less frequent types)	746 (N/A) (277 colonic, 267 central nervous system, 74 lymphoid, 72 breast and 56 upper aerodigestive tract tumors)
